# MIR31HG, a potential lncRNA in human cancers and non-cancers

**DOI:** 10.3389/fgene.2023.1145454

**Published:** 2023-08-10

**Authors:** Luxi Ruan, Jing Lei, Yihang Yuan, Huizi Li, Hui Yang, Jinyan Wang, Quanan Zhang

**Affiliations:** ^1^ Department of Oncology, The Affiliated Jiangning Hospital with Nanjing Medical University, Nanjing, Jiangsu, China; ^2^ Department of Oncology, Fudan University Shanghai Cancer Center, Shanghai, China

**Keywords:** MIR31HG, long non-coding RNA, expression, biological function, signalling pathways

## Abstract

Long non-coding RNAs have recently attracted considerable attention due to their aberrant expression in human diseases. LncMIR31HG is a novel lncRNA that is abnormally expressed in multiple diseases and implicated in various stages of disease progression. A large proportion of recent studies have indicated that MIR31HG has biological functions by triggering various signalling pathways in the pathogenesis of human diseases, especially cancers. More importantly, the abnormal expression of MIR31HG makes it a potential biomarker in diagnosis and prognosis, as well as a promising target for treatments. This review aims to systematically summarize the gene polymorphism, expression profiles, biological roles, underlying mechanisms, and clinical applications of MIR31HG in human diseases.

## 1 Introduction

Cancer is one of the leading causes of death worldwide with increasing incidence and mortality. Based on recent cancer statistics in 185 countries, 19.3 million new cases were diagnosed in 2021, and 10.0 million cancer patients died (Sung et al., 2021). In various types of cancers, abnormal expression of long non-coding RNAs (lncRNAs) can be detected, which is supposed to be related to proliferation, invasion, metastasis, and other biological aspects of cancer (Kopp and Mendell, 2018; Gyamfi et al., 2022; Vervoort et al., 2022). LncRNAs, more than 200 nucleotides in length, are non-coding RNA molecules that lack an open reading frame (Birney et al., 2007; Derrien et al., 2012). Unlike microRNAs(miRNAs), they cannot encode any protein but regulate chromatin dynamics, gene expression, growth, differentiation, and development due to their special length (Wang and Chang, 2011; Roberts et al., 2014). Several recent studies have indicated that lncRNAs play numerous roles in human malignant tumours (Cao et al., 2019; Zhou et al., 2021; Yang et al., 2022a).

LncRNA MIR31HG, also known as long non-coding HIF-1α coactivating RNA (LncHIFCAR) or LOC554202, is located in 9p21.3 with 2,166 bp in length and acts as a host gene for miR-31 in intron 2 (Augoff et al., 2012; Yan et al., 2018). We shed light on this recently-discovered lncRNA because several reports have shown that the lncRNA MIR31HG is aberrantly expressed in different cancers and affects numerous biological processes, including proliferation, metastasis, epithelial-mesenchymal transition (EMT), cellular senescence, and apoptosis in tumour development (Gupta et al., 2020; Tu et al., 2020). An increasing number of studies have also reported it can also participate in some signalling pathways (Zhang et al., 2021; Feng et al., 2022). Furthermore, the different functions performed by lncRNA MIR31HG depend on the tumour types and pathways involved. The complexity of these pathways presents great challenges but provides opportunities for the discovery of original cancer therapeutic targets and potential diagnostic biomarkers. Recently, more evidence has also shown that MIR31HG also participates in other diseases in addition to cancer, such as psoriasis, IgA nephropathy (IgAN), hirschsprung’s disease, rheumatoid arthritis (RA), and osteonecrosis of the femoral head (ONFH) (Cai et al., 2018; Gao et al., 2018; Yuan et al., 2020; Cao et al., 2021; Liu et al., 2022a).

This review aims to elaborate on the gene polymorphism, aberrant expression levels, biological roles, related mechanisms, and potential clinical applications of lncRNA MIR31HG in human diseases and concludes its function as a biomarker for the diagnosis, prediction, and treatment of human diseases.

## 2 MIR31HG gene polymorphisms

**FIGURE 1:**
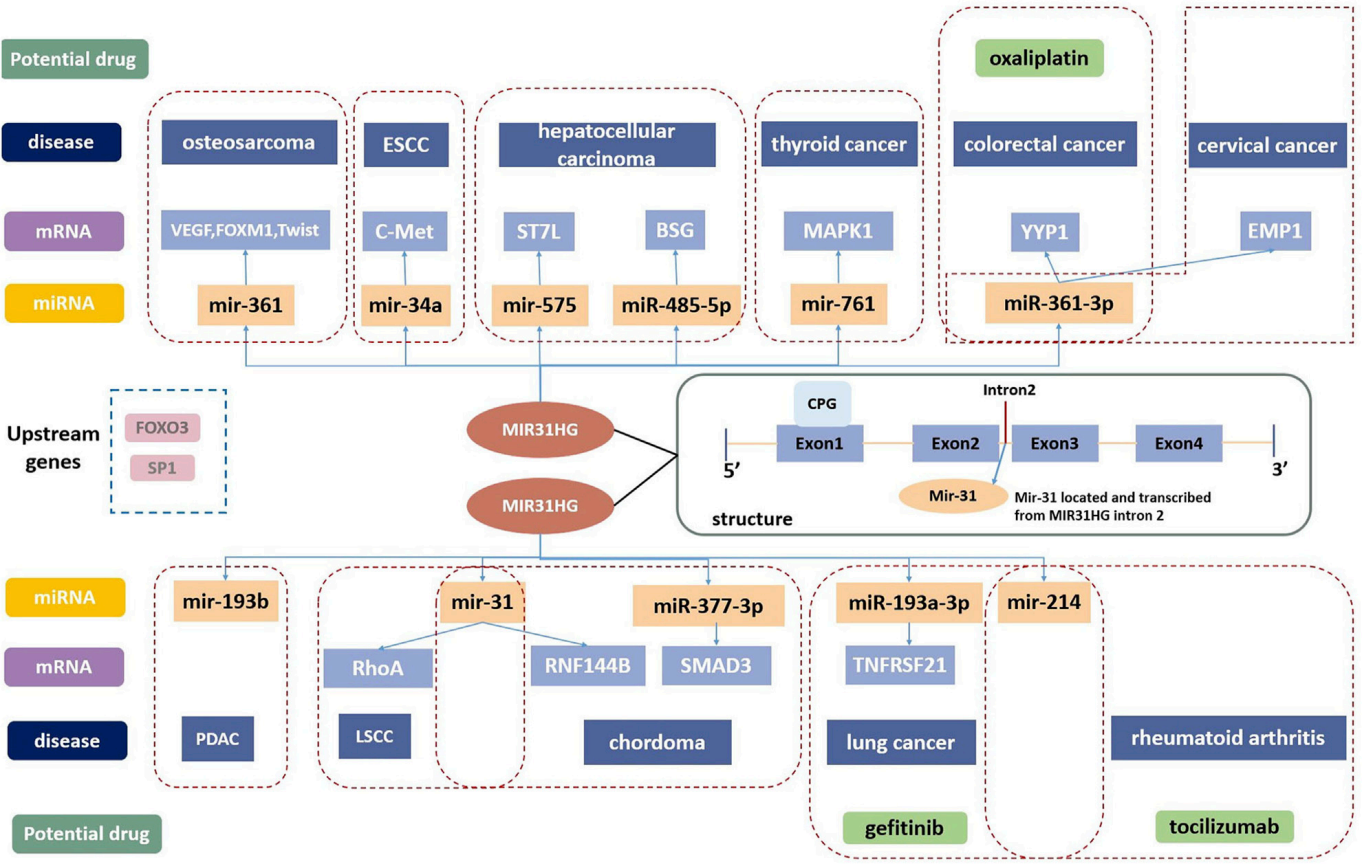
The structure of MIR31HG contains four exons and three junctions. Mir-31 is located in intron2. SP1 and FOXO3 are the upstream genes of MIR31HG. MIR31HG plays an important role in the lncRNA-miRNA-mRNA ceRNA network in disease development. MIR31HG can serve as a therapeutic target, and oxaliplatin, gefitinib and tocilizumab are related potential drugs for human diseases.

MIR31HG was first mentioned to participate in the translation process of mir31 and proved to be the host gene of mir31 in 2009 (Corcoran et al., 2009). MIR31HG contains four exons and three junctions, and mir31 is possibly located in intron2 (Augoff et al., 2012; Shih et al., 2017). A study in 2012 also assumed that there could be a large CPG island in its promoter, and showed the role of MIR31HG in promoting hypermethylation in human cancer (Augoff et al., 2012) (Figure 1).

Single nucleotide polymorphisms (SNPs) are involved in developing the role of specific genes in disease occurrence, and several recent analyses have revealed the supportive role of MIR31HG gene variations in the susceptibility of human diseases. Rs10965059, rs72703442, rs55683539, rs1332184, rs2181559, rs10965064, and rs2025327 were common SNPs used for MIR31HG polymorphism analyses (Daly et al., 2001). As an intronic variant of MIR31HG, rs10965059 is a crucial SNP because it was associated with susceptibility to various diseases including LDH, IgA nephropathy, steroid-induced osteonecrosis, and alcohol-induced osteonecrosis (Yuan et al., 2020; Liu et al., 2022a; Wang et al., 2022a; Hu et al., 2022). Aside from these non-cancers, MIR31HG polymorphisms also affect breast cancer (Wei et al., 2023). In Chinese women, three SNPS, rs72703442-AA, rs55683539-TT, and rs2181559-AA, were related to a lower risk of breast cancer (BC), while rs55683539 was considered the best risk-predictive single-locus model. According to estrogen receptor (ER) and progesterone receptor (PR) status analysis, rs79988146 was a relative gene variant for ER-positive and PR-positive breast cancer patients.

## 3 The expression level of MIR31HG in human diseases

**FIGURE 2:**
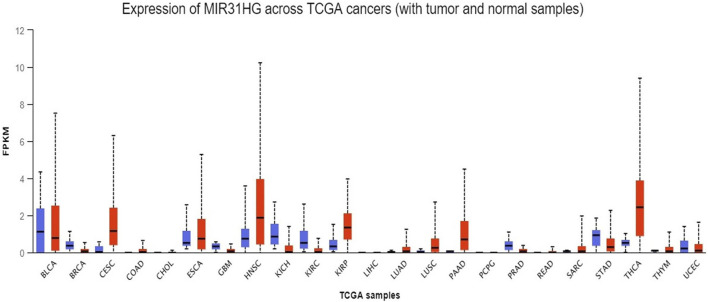
A pan-cancer analysis of MIR31HG expression in various cancers shows the overexpression of MIR31HG in BRCA, CESC, COAD, HNSC, LUAD, PAAD and THCA. Lower expression is found in KIRC and UCEC.

Considerable research in recent years has continually investigated MIR31HG expression levels in cancer, including gastric cancer (Nie et al., 2016; Lin et al., 2018), breast cancer (Augoff et al., 2012; Shi et al., 2014; Xin et al., 2021), lung cancer (Qin et al., 2018; Dandan et al., 2019; Zheng et al., 2019), colorectal cancer (Ding et al., 2015; Yang et al., 2016a; Li et al., 2018; Eide et al., 2019), bladder cancer (He et al., 2016; Sveen et al., 2020), head and neck squamous cell carcinoma (Ren et al., 2017; Wang et al., 2018a; Chu et al., 2020), osteosarcoma (Sun et al., 2019), melanoma (Xu and Tian, 2020) and other cancer types (Yang et al., 2016b; Shih et al., 2017; Feng et al., 2020; Li, 2020; Chen et al., 2022; Ko et al., 2022; Tu et al., 2022). A pan-cancer analysis of gene expression was performed by the UALCAN database using The Cancer Genome Atlas (TCGA) data to determine the expression of MIR31HG in human cancers (Figure 2). Furthermore, MIR31HG expression seemed to share a strong relationship with clinical characteristics in a number of cancers, such as tumour node metastasis (TNM), differentiation, distant metastasis, disease-free survival (DFS), and overall survival (OS) (Table 1). In addition to human cancer, in specific human non-cancer MIR31HG overexpression was able to be found in psoriasis (Gao et al., 2018), IgAN (Yuan et al., 2020), RA, and ONFH (Cao et al., 2021). However, it is downregulated in hirschsprung’s disease (Cai et al., 2018). MIR31HG significantly influenced this human non-cancer with pathological progression and clinical traits (Table 2).

**TABLE 1:** The expression, related functions, clinical features and related genes of MIR31HG in cancers.

NO	Cancer types	Regulation	Related functions	Clinical features	Related genes	Source of evidence	Ref
1	Gastric cancer	Up Down	Proliferation, migration	Poor prognosis	—	Cell line studies	Nie et al. (2016), Lin et al. (2018)
						Cell line, animal, and clinical studies	
2	Breast cancer	Up Down (TNBC)	Proliferation, migration	Tumour size, clinical stage	POLDIP2	Cell line, animal, and clinical studies	Augoff et al. (2012), Shi et al. (2014), Xin et al. (2021)
3	Lung cancer	Up	Proliferation, migration, invasion, metastasis, apoptosis, EMT	Clinical stage, TNM and OS	Mir-31 Mir-214	Cell line, animal, and clinical studies	Qin et al. (2018), Dandan et al. (2019), Zheng et al. (2019)
4	Colorectal cancer	Up Down	Proliferation, apoptosis, cell cycle, angiogenesis, glycolysis	OS and DSS Tumour size and pathologic stage	Mir-361-3p	Cell line, animal, and clinical studies	Ding et al. (2015), Yang et al. (2016a), Li et al. (2018), Eide et al. (2019)
5	Bladder cancer	Up Down	Proliferation, migration	TNM stage, OS and DFS	-	Cell line and clinical studies	He et al. (2016), Sveen et al. (2020)
6	ESCC	Up Down	Proliferation, migration, invasion	TNM, distant metastasis; differentiation, OS	Mir-34a/c-met	Cell line, animal, and clinical studies	Ren et al. (2017), Chu et al. (2020)
7	LSCC	Up	Proliferation, tumourigenesis, apoptosis	Advanced T category, OS, and RFS	Mir-31, RhoA HIF1A and P21	Cell line, animal, and clinical studies	Wang et al. (2018a)
8	Osteosarcoma	Up	Proliferation, invasion, migration, apoptosis, EMT	Metastasis	—	Cell line, animal, and clinical studies	Sun et al. (2019)
9	Melanoma	Up	Proliferation, invasion, migration	Metastasis, TNM stage	—	Cell line and clinical studies	Xu and Tian (2020)
10	Thyroid cancer	Up	Proliferation, invasion, migration, and apoptosis	Tumour size, lymph node metastasis	Mir-761	Cell line and clinical studies	Chen et al. (2022)
11	Cervical cancer	Up	Cell growth and invasion	Tumour size, lymph node metastasis and OS	Mir-361-3p,EMP1	Cell line, animal, and clinical studies	Li (2020)
12	Oral cancer	Up	Proliferation, invasion, and wound healing	Poor survival in stage IV, node metastasis in stage I-III	Mir-31, HIF-1α	Cell line, animal, and clinical studies	Shih et al. (2017), Tu et al. (2022)
13	PDAC	Up	Proliferation, invasion, apoptosis and EMT	DFS	Mir-193b	Cell line, animal, and clinical studies	Yang et al. (2016b), Ko et al. (2022)
14	NPC	Up	Proliferation, migration, and invasion	-	Mir-31/hroA	Cell line study	Feng et al. (2020)

**TABLE 2:** The expression, related functions, clinical features of MIR31HG in non-cancers.

NO	1	2	3	4	5
Non-cancer types	Psoriasis	IgA nephropathy	Hirschsprung’s disease	Rheumatoid arthritis	Osteonecrosis of femoral head
Regulation	Up	Up	Down	Up	Up
Related functions	Proliferation and cell cycle	—	Proliferation and migration	Proliferation, migration, inflammation	—
Clinical features	Diagnosis	Susceptibility	—	Treatment	Susceptibility
Source of evidence	Cell line study	Silico analysis	Cell line study	Cell line study	Silico analyses
Ref	Gao et al. (2018)	Yuan et al. (2020)	Cai et al. (2018)	Cao et al. (2021)	Liu et al. (2022a), Wang et al. (2022a)

### 3.1 The expression of MIR31HG in cancers

#### 3.1.1 Pan-cancer analysis of MIR31HG expression

First, to determine the expression of MIR31HG in human cancers, the UALCAN database was used to investigate the pancancer expression of MIR31HG (Chandrashekar et al., 2017). Based on the data from TCGA, overexpression of the lncRNA MIR31HG was discovered in breast invasive carcinoma (BRCA), cervical squamous cell carcinoma and endocervical adenocarcinoma (CESC), colon adenocarcinoma (COAD), head and neck squamous cell carcinoma (HNSC), lung adenocarcinoma (LUAD), pancreatic adenocarcinoma (PAAD) and thyroid carcinoma (THCA). On the contrary, lower MIR31HG expression was found in kidney renal clear cell carcinoma (KIRC) and uterine corpus endometrial carcinoma (UCEC). As this is a simple analysis only dependent on the database, we further examined the MIR31HG expression in various types of cancers by searching related experiments and clinical research.

#### 3.1.2 MIR31HG dysregulation in gastric cancer

Gastric cancer is the third most prevalent cancer all over the world, and its incidence and mortality have remained high in recent years (Thrift and El-Serag, 2020; Ajani et al., 2022; Yeoh and Tan, 2022). According to Nie’s research, a low expression level of MIR31HG was discovered in gastric cancer tissues compared with adjacent normal tissues. Its downregulated expression level was associated with larger tumour size, advanced pathological stage, and relatively poor prognosis (Nie et al., 2016). In contrast, MIR31HG was found to be overexpressed in gastric cancer in Lin’s study, especially in HGC27 and MGC-803 cell lines (Lin et al., 2018). On the one hand, MIR31HG played an important role in promoting cell proliferation and migration in MGC-803. On the other hand, in HGC27 cells, MIR31HG inhibited cell proliferation and migration, demonstrating the diverse functions of MIR31HG in different gastric cancer cell lines.

#### 3.1.3 MIR31HG dysregulation in breast cancer

Breast cancer accounts for nearly 25% of cancers in women and is currently the most lethal cancer in females worldwide (Bray et al., 2018; Wang et al., 2021a). The expression of lncRNA MIR31HG was upregulated in various types of breast cancers (Shi et al., 2014; Xin et al., 2021), while MIR31HG was downregulated in triple-negative breast cancer (Augoff et al., 2012). For the first time, Shi et al. (2014) showed that MIR31HG knockdown could result in diminished cell proliferation by modulating the G1–S checkpoint and apoptosis in breast cancer. Experimental evidence from another study also indicated that silencing MIR31HG can suppress breast cancer proliferation migration, and invasion by targeting polymerase (DNA-directed), delta interacting protein 2(POLDIP2) (Xin et al., 2021). MIR31HG expression was notably upregulated in 20 breast cancer tissues in two cell lines (MDA-MB-231 and MDA-MB435S) collected from BC patients who received surgical resection, contrasting with the former result in 2012 (Augoff et al., 2012). Functionally, executed as an oncogene, MIR31HG influences the apoptotic, proliferative, and invasive capabilities, as well as tumour size and clinical stage in breast cancer (Shi et al., 2014).

#### 3.1.4 MIR31HG dysregulation in lung cancer (LC)

It was estimated that nearly 2 million new lung cancer cases occurred and caused 1.76 million deaths in the past year, proposing the severity of LC worldwide (Brody, 2020; Thai et al., 2021). A great number of studies have demonstrated that MIR31HG overexpression in lung cancer tissues and related cell lines (A549, H2228, H1975, H1229) (Qin et al., 2018; Tu et al., 2020). A study on 152 tissue samples consisting of both lung adenocarcinoma and adjacent normal samples revealed that MIR31HG was related to advanced clinical staging and TNM stage (Qin et al., 2018). Zheng et al. (2019) performed research with 88 patients and demonstrated that the expression of MIR31HG also exhibited a close relationship with histological differentiation grade and lymph node metastasis in non-small cell lung cancer (NSCLC). In agreement with Zheng’s result, patients with MIR31HG overexpression are likely to have unfavourable OS (Dandan et al., 2019).

#### 3.1.5 MIR31HG dysregulation in colorectal cancer (CRC)

Colorectal cancer is the third most prevalent malignant tumour and the second most common cause of cancer deaths worldwide (Ciardiello et al., 2022; Sinicrope, 2022). For the first time, a study revealed that MIR31HG expression was lower than that in non-cancerous colorectal tissues, and another study from Yang et al. involving 178 samples also supported this finding (Ding et al., 2015; Yang et al., 2016a). Patients with lower MIR31HG appeared to have a worse outcome of OS and DFS in colorectal cancer (Yang et al., 2016a). Contrary to the former study, emerging evidence has shown an elevated level of MIR31HG in CRC tissues compared with adjacent tissues (Li et al., 2018; Wang et al., 2022b). Moreover, in association with miR-31-5p, patients with MIR31HG outlier status had shorter relapse-free survival (RFS), highlighting the prognostic role of MIR31HG in colorectal cancer (Eide et al., 2019). Furthermore, in an experiment on 221 patients treated with oxaliplatin, the high performance of MIR31HG was also associated with high DFS and OS rates (Li et al., 2018).

#### 3.1.6 MIR31HG dysregulation in bladder cancer

As the fourth most common malignancy in men and the most common malignancy in women, bladder cancer has been associated with high mortality and morbidity (Lenis et al., 2020). Growing evidence shows that MIR31HG expression is suppressed in bladder cancer and was consistent with the pan-cancer analysis (He et al., 2016). This study on 55 samples revealed that a lower level of MIR31HG was linked with advanced TNM stage, while another study indicated that in patients with the basal subtype, MIR31HG overexpression was discovered and correlated with poor OS and DFS (Sveen et al., 2020). The differentiation may be caused by different subtypes of bladder cancer in those studies.

#### 3.1.7 MIR31HG dysregulation in head and neck squamous cell carcinoma (HNSCC)

As one of the most lethal cancers in the world, esophageal squamous cell carcinoma (ESCC) accounts for nearly 90% of new esophageal cancer cases per year (Abnet et al., 2018; Yang et al., 2020). Based on research including 185 samples, lower MIR31HG expression was detected in ESCC tissues from patients with ESCC compared with the control. A lower MIR31HG level was associated with worse differentiation, advanced lymph node metastasis, positive distant metastasis, and poorer OS (Ren et al., 2017). In the other two studies, MIR31HG expression was found to be upregulated in ESCC (Sun et al., 2018; Chu et al., 2020). Fifty-three blood samples from ESCC patients and 39 blood samples from healthy people were collected in Sun’s study which proposed that higher expression of MIR31HG was positively related to advanced TNM stage and lymphatic metastasis. Moreover, they discovered that MIR31HG could distinguish ESCC patients from healthy individuals through ROC curve analysis in plasma, indicating the role of MIR31HG in diagnosis (Sun et al., 2018).

Laryngeal squamous cell carcinoma (LSCC) remains one of the most common HNSCCs and leads to approximately 20% of all cases (Mody et al., 2021). Indeed, MIR31HG expression was reported to be upregulated in LSCC. One study *in vitro* and *in vivo* revealed that combined with HIF1α (hypoxia-inducible factor 1α) and P21, MIR31HG significantly accelerated cell growth and impaired apoptosis. Furthermore, in this experiment, sixty LSCC patients were divided into two groups according to diverse expression levels, and patients with lower MIR31HG expression had significantly better OS and RFS. Therefore, more research with a larger sample size is demanded to clarify the function of MIR31HG in HNSCC (Wang et al., 2018a).

#### 3.1.8 MIR31HG dysregulation in osteosarcoma

Osteosarcoma, the most frequent type of primary malignant bone tumour, occurs mostly in adolescents and young adults (Kansara et al., 2014). With the development of curative therapy, wide resection surgery combined with chemo radiotherapy was applied and significantly improved the 5-year survival rate, but remains insufficient (Gill and Gorlick, 2021; Meltzer and Helman, 2021). An elevated level of MIR31HG was discovered in osteosarcoma samples. In detail, high expression of MIR31HG was associated with poor tumour stages and distant metastasis. Loss of MIR31HG further inhibited miR-361, which is a tumour suppressor that inhibits cell proliferation and migration. Except for miR-361, its downstream genes, such as vascular endothelial growth factor (VEGF), forkhead box protein 1(FOXM1), and Twist, were also suppressed in osteosarcoma cells, resulting in EMT and tumour growth (Sun et al., 2019). In summary, MIR31HG exerted oncogenic function by directly regulating miR-361 for tumour growth.

#### 3.1.9 MIR31HG dysregulation in melanoma

Malignant melanoma (MM) is known as a fatal malignant tumour caused by mutant melanocyte proliferation (Nassar and Tan, 2020). One study demonstrated that the expression of MIR31HG was significantly upregulated in melanoma. Abundant expression of MIR31HG was significantly correlated with lymph node metastasis, distal metastasis, and TNM stage, and served as a prognostic biomarker for MM (Xu and Tian, 2020).

#### 3.1.10 MIR31HG and other cancers

Based on research containing 57 papillary thyroid cancer (PTC) with a reference sample and four relative adjacent normal thyroid tissues, MIR31HG expression was correlated with M stage, N stage, and lymph nodes. For the first time, the research presented that higher expression of MIR31HG was connected with a higher level of immune infiltration in thyroid cancer (Chen et al., 2022). In cervical cancer, Li (2020) verified that MIR31HG silencing inhibited cervical cancer cell proliferation and invasion, whereas anti-miR-361-3p or overexpression of epithelial membrane protein 1 (EMP1) led to the opposite effect. Higher expression of MIR31HG can also be found in oral carcinoma, pancreatic ductal adenocarcinoma (PDAC), and nasopharyngeal carcinoma (NPC). The upregulated MIR31HG level in oral carcinoma was related to poor clinical outcomes and contributed to cancer progression (Shih et al., 2017). MIR31HG functions as an oncogene in PDAC, and the overexpression of MIR31HG is closely associated with poorer DFS in PDAC patients (Yang et al., 2016b; Ko et al., 2022). Increasing levels of MIR31HG enhanced NPC cell growth and metastasis, and could be inhibited by the regulation of mir-31 (Feng et al., 2020).

### 3.2 The expression of MIR31HG in non-cancers

#### 3.2.1 Psoriasis

Psoriasis is commonly regarded as a chronic immune-related papulosquamous skin disease and negatively affects the life quality of patients (Griffiths and Barker, 2007; Walter, 2022). Previous research indicated that interleukin-17A (IL-17A), interleukin-22(IL-22), tumor necrosis factor-α (TNF-α), and interleukin-1α (IL-1α) were pro-inflammatory cytokines in psoriasis and linked with NF-κB signalling activation (Guilloteau et al., 2010; De Simone et al., 2015). In psoriasis, MIR31HG was found to be elevated in psoriasis lesions and the upregulation of MIR31HG required IL-17A, IL-22, TNF-α, and IL-1α stimulation, demonstrating that nuclear factor kappa B inhibitor (NF-κB) signalling could be crucial crosstalk in psoriasis. Moreover, p65 targeted by specific small interfering RMA (siRNA) suppressed MIR31HG overexpression in human immortalized keratinocytes (HaCaT), consistent with the effect of BAY11-7082 (BAY, NF-κB) on cytokine-induced MIR31HG expression. Additionally, the inhibition of MIR31HG also dampened HaCaT cell proliferation (Gao et al., 2018).

#### 3.2.2 IgA nephropathy

IgA nephropathy (IgAN) is a chronic disease characterized by the deposition of IgA in the glomerular mesangium and is the most common glomerulonephritis worldwide (Saha et al., 2018; Pattrapornpisut et al., 2021). Expressed in human kidneys and participating in autoimmune diseases, more attention has been given to MIR31HG in recent years (Gupta et al., 2020). Through a study including 413 Chinese IgAN patients and 423 healthy people, several single nucleotide polymorphisms (SNPs) in MIR31HG, such as rs1332184 and rs55683539, were significantly associated with an increased risk of IgA nephropathy, creatively indicating that individuals with MIR31HG overexpression were likely to be more susceptible to IgAN (Yuan et al., 2020).

#### 3.2.3 Hirschsprung’s disease (HSCR)

Known as a congenital disease with a disorder of the enteric nervous system, HSCR commonly occurs among children (Langer, 2013; Das and Mohanty, 2017). Cai et al. (2018) made a hypothesis that miR-31, miR-31*, and their host gene MIR31HG may participate in the pathogenesis of hirschsprung’s disease. Then, they found that the downregulation of MIR31HG could suppress cell migration and proliferation through the MIR31HG-miR31/31*-ITIH5/PIK3CG pathway. While the downregulation of MIR31HG, miR-31, and miR-31* is not associated with the cell cycle and apoptosis.

#### 3.2.4 Rheumatoid arthritis

Rheumatoid arthritis (RA) is a chronic autoimmune disorder affected by multiple factors and mostly damages the joints (Smolen et al., 2016; Deane and Holers, 2021). Fibroblast-like synoviocytes (FLSs) the most abundant cell type in the joint synovium become inflamed and even invade bones in RA (Tu et al., 2018). Using specific siRNA to downregulate MIR31HG expression promoted cell proliferation, migration, and inflammation in RA-FLS. For clinical applications, tocilizumab could suppress RA-FLSs inflammation by targeting MIR31HG, indicating the protective role of MIR31HG in RA (Cao et al., 2021)

#### 3.2.5 Osteonecrosis of the femoral head (ONFH)

Osteonecrosis of the femoral head is a complicated hip disability characterized by bone necrosis and usually occurs in young adults with an average age of 30–50 years old (Petek et al., 2019). Steroid use, alcohol abuse, and smoking are defined as common non-traumatic leading causes of ONFH (Wang et al., 2018b). Since previous studies have discovered that abnormal lncMIR31HG expression can affect osteogenic differentiation and that its polymorphism is correlated with radius bone mineral content in boys (Chesi et al., 2015). Two studies further investigated the MIR31HG gene variant in steroid-induced and alcohol-induced osteonecrosis. In one study including 708 Chinese Han volunteers, both age and sex were related to MIR31HG polymorphisms, and MIR31HG-rs10965059 was associated with a lower risk of bilateral steroid-induced osteonecrosis (Wang et al., 2022a). MIR31HG-rs10965059 and MIR31HG-rs10965064 were strongly associated with a lower risk of disease occurrence especially in patients over 40 years old and rs10965059 served as a protective gene in alcohol-induced osteonecrosis (Liu et al., 2022a). However, the precise mechanism by which MIR31HG participates in pathogenesis remains unclear.

## 4 The biological role of MIR31HG in human diseases

### 4.1 MIR31HG and the cell cycle

LncRNA MIR31HG has been revealed to control the cell cycle during human disease development. For instance, Ding et al. (2015) indicated that overexpression of MIR31HG may promote colorectal cancer cell arrest in the G0/G1 phase and induce apoptosis through the activation of specific caspase cleavage cascades. A study performed by Yang et al. (2016b) demonstrated that the knockdown of MIR31HG significantly induced G1/S arrest in pancreatic ductal adenocarcinoma (PDAC), whereas enhanced expression of MIR31HG had the opposite effects. Qin et al. (2018) also showed that downregulation of MIR31HG inhibited the proliferation of lung adenocarcinoma cells and blocked the G0/G1 to S-phase transition in cell cycle progression, but had no effect on cell apoptosis. In psoriasis, the silencing of lncRNA MIR31HG induced cell cycle arrest in the G2/M phase potentially via the mediation of siRNA (Gao et al., 2018).

### 4.2 MIR31HG and the EMT

Epithelial-to-mesenchymal transition (EMT) is a biological process by which polarized epithelial cells are transformed into highly motile mesenchymal cells (Thiery and Sleeman, 2006). Numerous types of research have proven that EMT plays an essential role in tumour progression and metastasis (Thiery, 2002; Hugo et al., 2007). Moreover, lncRNAs including HOTAIR, H19, ATB, and MIR100HG are commonly known as regulators that are involved in the EMT process (Dhamija and Diederichs, 2016). In particular, the lncRNA MIR100HG also acts as a host gene for miRNAs like MIR31HG, and a recent study emphasized its function as a positive regulator in EMT to advance colorectal cancer cell evasion and metastasis (Liu et al., 2022b).

Recent advances in sequencing technology have revealed that the lncRNA MIR31HG may serve as an oncogenic regulator in PDAC by promoting EMT. Transforming growth factor β (TGFβ) signalling plays dual roles in cancer progression, especially in the later stages of tumourigenesis. The high amounts of TGFβ secreted by cancer cells promote tumour progression by inducing EMT (Zhang et al., 2017a). Thus, Ko et al. (2022) found that the enrichment of TGFβ signalling in PDAC and the presence of MIR31HG enhanced TGFβ-induced EMT, suggesting that MIR31HG could serve as an oncogene in PDAC. Furthermore, as an epithelial marker, E-cadherin levels were found to be elevated, while mesenchymal signs such as Vimentin and transcription factors like Twist1 were remarkably downregulated in NSCLC cells through the silencing of MIR31HG (35). Patients with MIR31HG overexpression tend to share higher TGFβ and EMT gene expression in colorectal cancer, implying a potential relationship between MIR31HG and the EMT gene signature in other cancer types (Eide et al., 2019).

### 4.3 MIR31HG and senescence

Cellular senescence is an irreversible arrest of cell proliferation that occurs when cells experience potentially oncogenic stress. Because of its limitation on cellular fission, it is regarded as an anticancer mechanism (Kuilman et al., 2010; Storer et al., 2013). Cellular senescence mainly relies on two tumour suppressor pathways: p14ARF/p53 and p16INK4A/pRB. Several lncRNAs are involved in cellular senescence. By modulating SAFA-PANDA-PRC communication, lncRNA PANDA restricted the cell proliferative state and dampened cellular senescence. Conversely, the loss of PANDA promoted senescence, serving as an opportunity for dropping out of senescence (Kotake et al., 2011; Puvvula et al., 2014). Recent research has shown that the lncRNA MIR31HG is associated with oncogene-induced senescence (OIS) through various targets.

From Marta Montes’s study in 2015, MIR31HG was upregulated in OIS, and the knockdown of MIR31HG could induce a senescence phenotype. Moreover, this research has also demonstrated that the role of nuclear MIR31HG in senescence relies on p16INK4A since depleting p16INK4A can eliminate these effects. On the contrary, no evidence suggests that MIR31HG has a relationship with the p14ARF/p53 pathway in modulating senescence. The senescence-associated secretory phenotype (SASP) has been shown to either restrain or enhance tumour progression. Therefore, it is promising to find certain factors that could affect SASP without influencing the tumour-suppressive effects related to senescence at the same time for advanced therapies (Montes et al., 2015). In 2021, Marta Montes further discovered that cytoplasmic MIR31HG modulated the expression and secretion of SASP-related components by interacting with YBX1 to induce IL1A translation. In conclusion, the lncRNA MIR31HG both promotes and suppresses senescence, and these different effects mainly rely on MIR31HG localization, further indicating that inhibition of MIR31HG could potentially be used in senescence-related pathology therapies (Montes et al., 2021).

### 4.4 MIR31HG and cell differentiation

Numerous studies have shown the relevance of lncRNAs in cell differentiation (Richart et al., 2022; Wu et al., 2022). For example, the overexpression of lncRNA Snhg6 was found in tumour-derived myeloid-derived suppressor cells (MDSCs) and contributed to MDSC differentiation by inhibiting the ubiquitination of EZH2 (Lu et al., 2021). Recent studies have explored whether lncRNA MIR31HG participates in cell differentiation, especially in adipocyte and osteogenic differentiation.

According to Huang’s research, inhibition of MIR31HG suppressed adipocyte differentiation of human adipose-derived stem cells (hASCs) via histone modification of fatty acid binding protein 4 (FABP4) (Huang et al., 2017). FABP4 is a kind of protein that is highly expressed in adipose tissue and can be targeted for metabolic disease treatment (Haunerland and Spener, 2004). This research suggests potential determinants of the applications of MIR31HG in obesity and other disorders. Apart from participating in adipocyte differentiation, MIR31HG may play an important role in osteogenic differentiation. HASCs are a type of mesenchymal stem cell (MSC) capable of bone regeneration and repair, making them attractive in bone tissue engineering (Tapp et al., 2009). Upregulated by inflammatory cytokines such as TNF-α and IL-17 by the NF-κB Signalling pathway, MIR31HG inhibited osteoblast differentiation of hASCs. In contrast, the knockdown of MIR31HG could promote bone formation, demonstrating that the inhibition of MIR31HG benefits bone regeneration and relieves inflammation (Jin et al., 2016).

## 5 Mechanism of MIR31HG-mediated biological function in human diseases

### 5.1 The Wnt/β-catenin signalling pathway

Wnt/β-catenin is a family of proteins that play an important role in controlling embryonic and organ development, as well as cancer progression (Clevers and Nusse, 2012). The Wnt/β-catenin pathway is able to confine the transcription of downstream genes by activating the expression of β-catenin (Chatterjee et al., 2022). It was reported that a high level of MIR31HG contributes to the progression of a variety of cancers by activating canonical Wnt signaling, which is also recognized as the Wnt/β-catenin signalling pathway.

**FIGURE 3:**
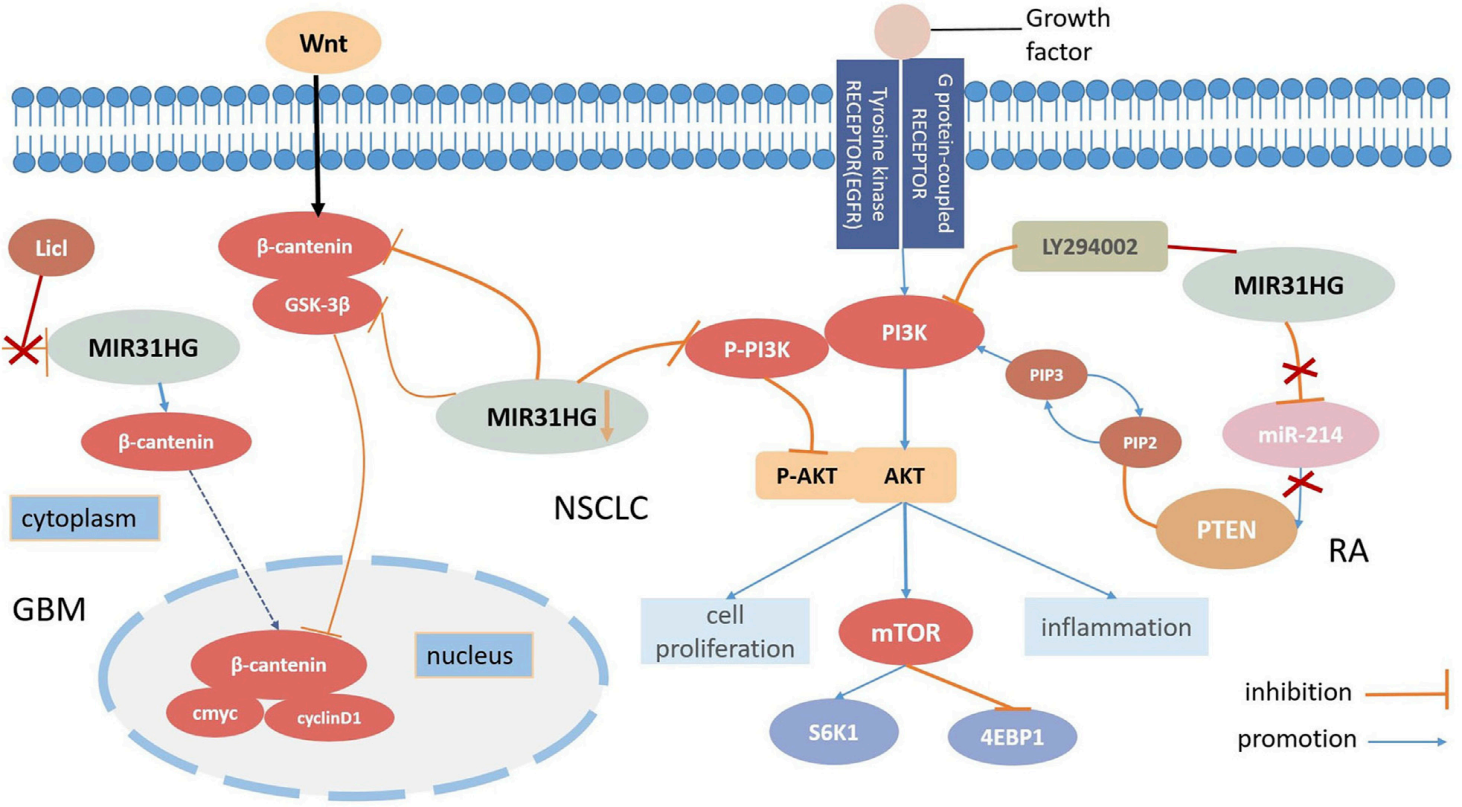
MIR31HG mediates biological function by participating the Wnt-β-catenin pathway and the AKT pathway in human diseases.

In contrast to some lncRNAs, MIR31HG is located mainly in the cytoplasm (Montes et al., 2015). Zheng et al. elucidated that the downregulation of MIR31HG depressed the Wnt/β-catenin signalling pathway via the inhibition of GSK3β and β-catenin expression levels but induced p-GSK3β overexpression in NSCLC cells. On the contrary, MIR31HG was demonstrated to enhance cell proliferation and invasion by activating this pathway in NSCLC (Zheng et al., 2019). The activation of the Wnt/β-catenin pathway was also found in glioblastoma (GBM) progression (Zhang et al., 2021). Triggered by STAT1, MIR31HG could transcribe β-catenin from the cytoplasm into the nucleus, and the Wnt/β-catenin pathway activator LiCl was utilized to invert both the ability to inhibit proliferation and impress apoptosis caused by MIR31HG knockdown in glioblastoma (Zhang et al., 2021). Therefore, we can safely conclude that the activation of the Wnt/β-catenin signaling pathway regulated by MIR31HG enhances cell growth in glioblastoma (Figure 3).

### 5.2 The AKT pathway

AKT kinases which are also called protein kinase B(PKB), are signalling molecules of cell growth and differentiation and the AKT pathway is commonly involved in inhibiting cell apoptosis and stimulating cell proliferation (Mundi et al., 2016; Stratikopoulos and Parsons, 2016). Among those AKT pathways, the PI3K (phosphatidylinositol 3-kinase)/AKT signaling pathway is one of the most prevalent and distinctive pathways related to growth, which modifies biological mechanisms in human diseases. For instance, the PI3K/AKT/mTOR (mammalian target of rapamycin) signalling pathway exerts an essential role in the tumourigenesis of malignant cancers (Barrett et al., 2012; Alzahrani, 2019). Moreover, phosphatase and tensin homolog (PTEN) is an important tumour suppressor that can dephosphorylate phosphatidylinositol (Birney et al., 2007; Gyamfi et al., 2022; Vervoort et al., 2022)-triphosphate (PIP3) to phosphatidylinositol 4,5-biphosphate (PIP2) and suppress the PI3K/AKT signalling pathway in various cancers (Bonneau and Longy, 2000). Many lncRNAs employ a cooperative effect as fine-tuners on both tumour inhibition and oncogenesis (Ghafouri-Fard et al., 2021). LncRNA FER1L4 evokes cell cycle arrest and AB073614 stimulates proliferation and hampers apoptosis; both of these processes are regulated by the AKT signalling pathway (Wang et al., 2019). Recently, several types of research have shown that the lncRNA MIR31HG may have a relationship with the AKT signalling pathway in various diseases (Figure 3).

In the case of NSCLC, Wang discovered that the overexpression of MIR31HG could not directly modify all epidermal growth factor receptor (EGFR), PI3K, or AKT levels but could affect the expression levels of P-EGFR, P-PIP3 and P-AKT. Research has elucidated that MIR31HG influences NSCLC cell proliferation, apoptosis, and the cell cycle by driving the EGFR/PI3K/AKT pathway, and even contributing to gefitinib resistance (Wang et al., 2017). The downregulation of P-PIP3 and P-AKT can also be seen in nasopharyngeal carcinoma (NPC) through MIR31HG silencing. In this study, silencing of MIR31HG decreased cell proliferation but promoted apoptosis; however, 740Y-P, a PI3K agonist successfully reversed this process. A positive correlation between MIR31HG and AKT expression levels was further demonstrated, suggesting that MIR31HG enhanced cell proliferation and induced apoptosis in NPC at the same time through the PI3K/AKT signalling pathway (Feng et al., 2022). RA-FLSs share several tumour cell-like characteristics with cancers, and a number of studies have demonstrated that PTEN may participate in RA-FLS formation. According to Cao’s study, MIR31HG and PTEN played roles as suppressive targets in RA-FLS inflammation regulated by mir214 and further motivated the AKT signalling pathway. Furthermore, the attachment of the PI3K inhibitor LY294002 remedied RA-FLS hyperinflammation induced by the loss of MIR31HG, suggesting that MIR31HG may serve as an upstream target for the AKT signalling pathway (Cao et al., 2021). In conclusion, MIR31HG inhibited proliferation, migration, and inflammation via regulation of the downstream miR-214-PTEN-AKT pathway. According to the gene set enrichment analysis (GSEA), MIR31HG may also contribute to colorectal cancer invasion and metastasis by modulating the PI3K-AKT-mTOR-signalling pathway (Wang et al., 2022b).

### 5.3 The lncRNA-miRNA-mRNA ceRNA network

Apart from the two pathways described above, MIR31HG also acts on a series of targets and participates in other pathways. The competing endogenous RNA (ceRNA) regulatory network is the main mechanism of MIR31HG in cancer development (Li and Zhan, 2019). Through ceRNA mechanisms, MIR31HG could act as a miRNA sponge to regulate the expression of downstream messenger RNAs (mRNAs), and affect tumour progression (Figure 1).

MIR31HG serves as an oncogene in cervical carcinoma by acting as a sponge for miR-361-3p to modulate the miRNA target EMP1 (Li, 2020). In colorectal cancer, miR-361-3p was inhibited by MIR31HG which thereby increased the YY1 level, contributing to tumour progression (Guo et al., 2021). By sponging miR-34a, MIR31HG enhances the expression of c-Met and promotes the development of ESCC (Chu et al., 2020). In hepatocellular carcinoma, FOX3-induced lncRNA LOC554202 upregulated BSG by competitively binding to miR-485-5p and promoted tumour progression (Yang et al., 2022b). In addition, MIR31HG inhibited hepatocellular carcinoma (HCC) proliferation and metastasis by directly binding miR-575 to positively modulate the expression of ST7L (Yan et al., 2018). MiR-31 is suppressed by the lncRNA MIR31HG in various cancers, including chordoma and LSCC. MIR31HG plays a crucial role in the progression of chordoma by indirectly promoting RNF144B via miR-31 (Ma et al., 2017). In LSCC, MIR31HG acted as an oncogene directly by inhibiting miR-31 expression and promoting its target gene RhoA expression (Yang et al., 2018). The modulation function of MIR31HG in regulating MAPK1 expression was completed by competitively sponging miR-761 in thyroid cancer (Peng et al., 2022). Furthermore, overexpression of MIR31HG promoted tumour growth in osteosarcoma cells by downregulating miR-361 expression and elevating the expression of VEGF, FOXM1 and Twist, which are target genes of miR-361 (Sun et al., 2019). Sp1-activated-MIR31HG advanced cell migration and invasion by directly binding to miR-214 in NSCLC (Dandan et al., 2019). According to the latest research from Mo’s team, MIR31HG serves as an oncogene by targeting mir-193a-3p and positively enhances recombinant tumor necrosis factor receptor superfamily, member 21 (TNFRSF21) expression in lung adenocarcinoma (Mo et al., 2022). In pancreatic ductal adenocarcinoma, lncMIR31HG exhibited oncogenic properties through the downregulation of miR-193b (Yang et al., 2016b).

### 5.4 Interaction with hypoxia-inducible factor 1α (HIF-1α)

Hypoxia-inducible factor 1α (HIF-1α) is the HIF-1 transcription factor and can modulate the expression of hypothetical genes (LaGory and Giaccia, 2016). As hypoxia induction can cause tumour metastasis and lead to worse prognosis, a number of studies have demonstrated the role of the HIF-1α pathway in cancer development in recent years (Zhou et al., 2015; Wu et al., 2017). In a recent review, upregulated lncRNAs including HOTAIR, H19, and MALAT1 were found and concluded to be hypoxia-responsive lncRNAs in cancers (Wang et al., 2021b). Similar to LncH19, lncMIR31HG was also induced by hypoxia and was subsequently named lncHIFCAR (long non-coding HIF-1α coactivating RNA) (Shih et al., 2017).

Through a previous integrated analysis of the expression of lncRNA-mRNA in advanced LSCC, lncMIR31HG was positively related to HIF-1α (Wang et al., 2018a). Western blotting further proved that the knockdown of MIR31HG inhibited HIF-1α expression and increased P21 expression. Moreover, in oral cancer, MIR31HG directly binds with HIF-1α and forms a special complex (Shih et al., 2017). This complex recruits HIF-1α and its coactivator p300, contributing to the overexpression of MIR31HG and activation of the HIF-1α pathway. Surprisingly, even under normoxic conditions, MIR31HG still enhanced the target genes of HIF-1α, indicating that MIR31HG could serve as a HIF-1α coactivator. Unfortunately, studies on lncMIR31HG and HIF-1α were only conducted before 2020 and the specific mechanisms were unclear.

## 6 MIR31HG as a potential biomarker in human diseases

### 6.1 MIR31HG as a diagnostic biomarker

Multiple kinds of research have elucidated that long non-coding RNAs participate in human diseases (Yang et al., 2022a; Hao et al., 2023). In view of the aberrant expression level, wide functions and gradually displayed underlying mechanisms, we will further discuss that lncRNA MIR31HG acts as a potential diagnostic biomarker in human cancer and non-cancers.

Differential expression of MIR31HG in specific tissues in cancers helps to distinguish diseased tissues from normal tissues, indicating that MIR31HG could be a potential biomarker for early cancer diagnosis (Ren et al., 2017; Yan et al., 2018). Apart from cancer diagnosis, the latest research focuses on the diagnostic role of MIR31HG in human non-cancers. Genetic factors play a crucial role in the development of lumbar disc herniation (LDH), which is a common spinal disease that poses a great threat to human health both worldwide and in China (Deyo and Mirza, 2016; Zhang et al., 2017b). By performing a multifactor dimensionality reduction (MDR) analysis, individuals with the MIR31HG polymorphism rs10965059 were found to be at great risk of LDH providing the possibility for early screening, prevention and diagnosis of Chinese Han LDH high-risk populations (Hu et al., 2022).

### 6.2 MIR31HG as a prognostic biomarker

**FIGURE 4:**
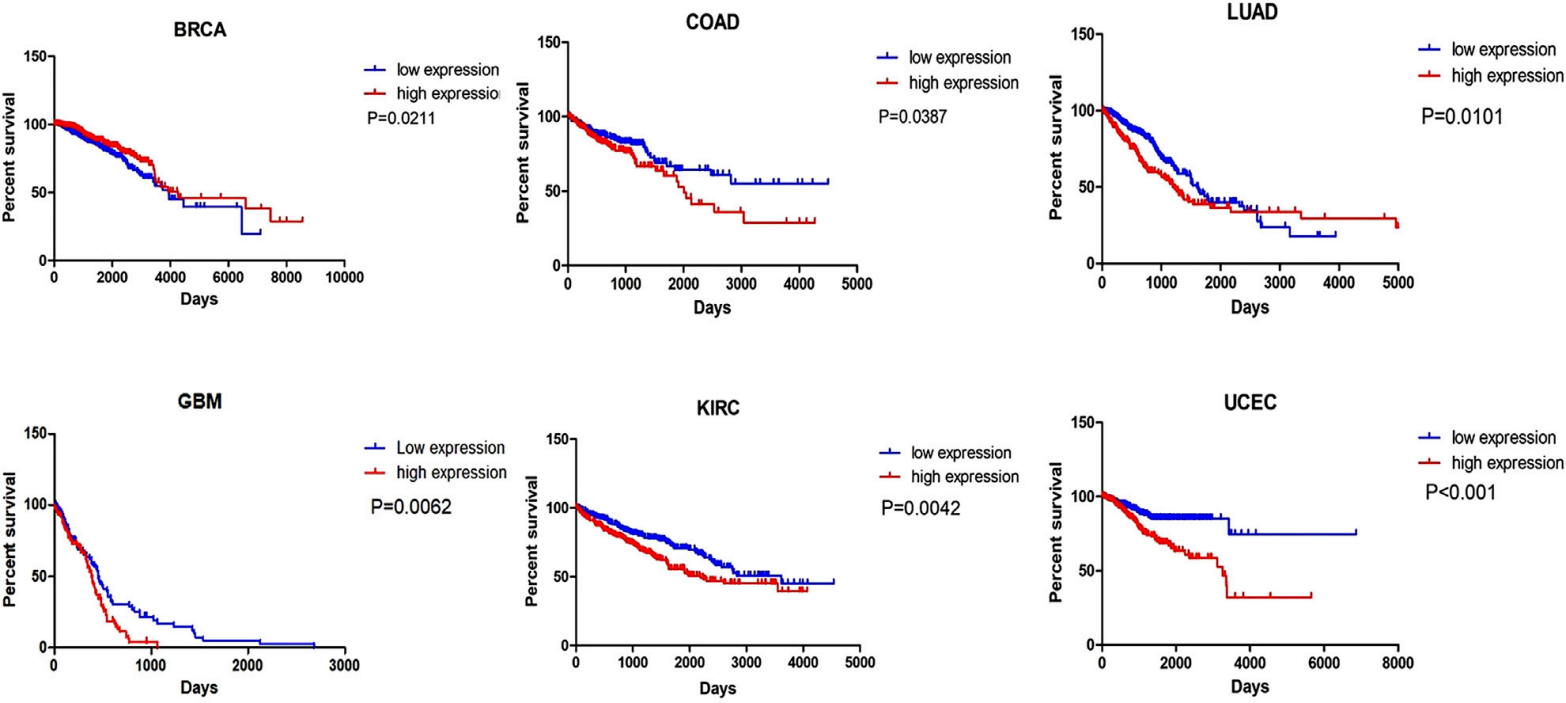
The survival analysis of MIR31HG indicates that MIR31HG is related to the OS of patients in six types of cancers. And in BRCA, different from other cancers, MIR31HG overexpression is associated with a better outcome.

Numerous studies have revealed that MIR31HG is positively related to clinicopathological features, including tumour size, clinical stage, TNM stage, advanced T category, lymph node metastasis, distant metastasis, overall survival, and progression-free survival (Table1). These clinical features elucidate the possibility that MIR31HG could serve as a prognostic biomarker in human cancer. For example, downregulation of MIR31HG in colorectal cancer was significantly associated with TNM stage, histologic grade, and lymph node metastasis, indicating that MIR31HG expression was linked with poor prognosis in CRC (Yang et al., 2016a). In oral squamous cell carcinoma, MIR31HG overexpression is related to a worse survival tendency in stage IV diseases (Tu et al., 2022). Furthermore, we conducted a survival analysis of MIR31HG in 21 kinds of human cancers using the Oncolnc database (http://www.oncolnc.org) (Figure 4). All data used for analysis in this database came from TCGA, and a log-rank *p*-value less than 0.05 was viewed as a great difference. LncRNA MIR31HG has a relationship with the OS of patients with breast invasive carcinoma (BRCA), lung cancer, colorectal cancer, glioblastoma, kidney renal clear cell carcinoma (KIRC) and uterine corpus endometrial carcinoma (UCEC). In breast cancer, a higher level of MIR31HG was associated with longer OS, while in other cancers, its overexpression was related to a worse outcome. This analysis suggested the potential role of MIR31HG in predicting cancer prognosis. Nevertheless, research has yet to systematically investigate the association between MIR31HG and human non-cancer.

### 6.3 MIR31HG as a therapeutic reagent

Drug resistance is a leading obstacle to human disease treatment (Vasan et al., 2019). With further research on the mechanisms involved in lncRNA-related disease pathogenesis, lncMIR31HG could be a promising biomarker for specific disease treatments. In NSCLC patients with EGFR mutations, LOC554202 reduced the sensitivity of NSCLC cells to gefitinib and promoted gefitinib resistance by regulating mir31 expression (He et al., 2019). For colorectal patients who have been treated with oxaliplatin-based chemotherapy, higher expression levels of LOC554202 were associated with DFS and OS rates. This result suggested that LOC554202 may be a potential marker for evaluating the outcome of colorectal cancer therapy (Li et al., 2018).

Tissue inflammation is one of the classic symptoms of RA (Buch et al., 2021). For treating moderate to severe RA in adults, either utilizing tocilizumab alone or in combination with other doses was viewed as a common therapy. MIR31HG, targeted by tocilizumab, was indicated to suppress RA-FLS inflammation and become a potential therapeutic target for RA in Cao’s study (Cao et al., 2021).

## 7 Conclusion and perspective

Plenty of existing evidence shows that lncRNA play an important role in the pathogenesis of human diseases. With further study of lncRNAs in human cancer, it is worth exploring and concluding the role of lncRNAs in tumour development. In this review, we summarize the current research on the role of MIR31HG in human cancer and non-cancer. MIR31HG gene polymorphism is associated with susceptibility to several diseases and plays an oncogenic role or acts as a tumour suppressor by regulating tumour cell proliferation, apoptosis, the cell cycle, EMT, and senescence. MIR31HG also participated in the differentiation of hASCs in non-cancer. These effects are realized by diverse mechanisims, such as the Wnt/β pathway, the AKT pathway, the lncRNA-miRNA-mRNA ceRNA network and interaction with HIF-1α (Figure 3).

MIR31HG, upregulation or downregulation, may act as a biomarker for the prognosis and diagnosis of cancer. Moreover, the dysregulation of MIR31HG in various cancers is significantly related to important clinical features including tumour size, TNM staging, histological grade, OS, and DFS (Table1). For human disease treatment, MIR31HG also serves as a therapeutic target for different diseases including NSCLC, colorectal cancer, and RA.

In conclusion, the lncRNA MIR31HG participates in the pathogenesis of human diseases and has great potential for clinical application by functioning as a diagnostic or prognostic biomarker and therapeutic target in human diseases.

However, there are some limitations in those studies. First, controversy still exists regarding the specific MIR31HG expression level in certain cancers, such as breast cancer, colorectal cancer, bladder cancer and ESCC. These expression differences may be the consequence of the diverse cell lines used and the specific patients selected. Second, in cancers such as PDAC and NPC, only one or two studies have depicted the role of MIR31HG in their development. The lack of multiple experiments can mislead our cognitions and obtain controversial results.

Although it has been shown that MIR31HG is also dysregulated in non-cancers, those studies are merely limited to cell line studies and *in silico* analysis. More animal experiments and clinical research are needed in the future. To date, MIR31HG has been shown to participate in only five human non-cancers, far less than its participation in cancers. Whether lncMIR31HG plays an important role in human non-cancer pathogenesis needs more solid support in wider types of diseases.

Functional experiments show that lncMIR31HG affects human diseases through four main mechanisms. However, the related mechanistic pathways remain in their primary stage, especially in the WNT pathway. Moreover, current studies mostly focus on the investigation of MIR31HG downstream regulators, and other studies on its upstream genes or regulators should be performed in the future. In addition, the lncRNA MIR31HG is correlated with drug resistance and treatment outcomes in cancers. However, its clinical value in non-cancers is not clear. Consequently, further high-equality experiments and credible clinical research are required to explore the latent value underlying MIR31HG in disease pathogenesis and treatment.

## References

[B1] Abnet C. C. Arnold M. Wei W. Q. (2018). Epidemiology of esophageal squamous cell carcinoma. Gastroenterology 154 (2), 360–373. Epub 2017/08/22. 10.1053/j.gastro.2017.08.023 28823862 PMC5836473

[B2] Ajani J. A. D'Amico T. A. Bentrem D. J. Chao J. Cooke D. Corvera C. (2022). Gastric cancer, version 2.2022, nccn clinical practice guidelines in oncology. J. Natl. Compr. Cancer Netw. JNCCN 20 (2), 167–192. Epub 2022/02/08. 10.6004/jnccn.2022.0008 35130500

[B3] Alzahrani A. S. (2019). Pi3k/Akt/Mtor inhibitors in cancer: at the bench and bedside. Seminars cancer Biol. 59, 125–132. Epub 2019/07/20. 10.1016/j.semcancer.2019.07.009 31323288

[B4] Augoff K. McCue B. Plow E. F. Sossey-Alaoui K. (2012). Mir-31 and its host gene lncrna Loc554202 are regulated by promoter hypermethylation in triple-negative breast cancer. Mol. cancer 11, 5. Epub 2012/02/01. 10.1186/1476-4598-11-5 22289355 PMC3298503

[B5] Barrett D. Brown V. I. Grupp S. A. Teachey D. T. (2012). Targeting the pi3k/akt/mtor signaling Axis in children with hematologic malignancies. Paediatr. drugs 14 (5), 299–316. Epub 2012/08/01. 10.2165/11594740-000000000-00000 22845486 PMC4214862

[B6] Birney E. Stamatoyannopoulos J. A. Dutta A. Guigó R. Gingeras T. R. Margulies E. H. (2007). Identification and analysis of functional elements in 1% of the human Genome by the encode pilot project. Nature 447 (7146), 799–816. Epub 2007/06/16. 10.1038/nature05874 17571346 PMC2212820

[B7] Bonneau D. Longy M. (2000). Mutations of the human pten gene. Hum. Mutat. 16 (2), 109–122. Epub 2000/08/03. 10.1002/1098-1004(200008)16:2<109:aid-humu3>3.0.co;2-0 10923032

[B8] Bray F. Ferlay J. Soerjomataram I. Siegel R. L. Torre L. A. Jemal A. (2018). Global cancer statistics 2018: globocan estimates of incidence and mortality worldwide for 36 cancers in 185 countries. CA a cancer J. Clin. 68 (6), 394–424. Epub 2018/09/13. 10.3322/caac.21492 30207593

[B9] Brody H. (2020). Lung cancer. Nature 587 (7834), S7. Epub 2020/11/20. 10.1038/d41586-020-03152-0 33208969

[B10] Buch M. H. Eyre S. McGonagle D. (2021). Persistent inflammatory and non-inflammatory mechanisms in refractory rheumatoid arthritis. Nat. Rev. Rheumatol. 17 (1), 17–33. Epub 2020/12/10. 10.1038/s41584-020-00541-7 33293696

[B11] Cai P. Li H. Huo W. Zhu H. Xu C. Zang R. (2018). Aberrant expression of lncrna-mir31hg regulates cell migration and proliferation by affecting mir-31 and mir-31* in hirschsprung's disease. J. Cell Biochem. 119 (10), 8195–8203. 10.1002/jcb.26830 29626357

[B12] Cao H. L. Liu Z. J. Huang P. L. Yue Y. L. Xi J. N. (2019). Lncrna-rmrp promotes proliferation, migration and invasion of bladder cancer via mir-206. Eur. Rev. Med. Pharmacol. Sci. 23 (3), 1012–1021. 10.26355/eurrev_201902_16988 30779067

[B13] Cao L. Jiang H. Yang J. Mao J. Wei G. Meng X. (2021). Lncrna Mir31hg is induced by tocilizumab and ameliorates rheumatoid arthritis fibroblast-like synoviocyte-mediated inflammation via mir-214-pten-akt signaling pathway. Aging (Albany NY) 13 (21), 24071–24085. Epub 2021/11/11. 10.18632/aging.203644 34753831 PMC8610144

[B14] Chandrashekar D. S. Bashel B. Balasubramanya S. A. H. Creighton C. J. Ponce-Rodriguez I. Chakravarthi B. (2017). Ualcan: a portal for facilitating tumor subgroup gene expression and survival analyses. Neoplasia (New York, NY) 19 (8), 649–658. Epub 2017/07/22. 10.1016/j.neo.2017.05.002 PMC5516091 28732212

[B15] Chatterjee A. Paul S. Bisht B. Bhattacharya S. Sivasubramaniam S. Paul M. K. (2022). Advances in targeting the wnt/Β-catenin signaling pathway in cancer. Drug Discov. today 27 (1), 82–101. Epub 2021/07/13. 10.1016/j.drudis.2021.07.007 34252612

[B16] Chen C. Qin L. Xiao M. F. (2022). Long noncoding rna Loc554202 predicts a poor prognosis and correlates with immune infiltration in thyroid cancer. Comput. Math. Methods Med. 2022, 3585626. 10.1155/2022/3585626 35265169 PMC8901293

[B17] Chesi A. Mitchell J. A. Kalkwarf H. J. Bradfield J. P. Lappe J. M. McCormack S. E. (2015). A trans-ethnic genome-wide association study identifies gender-specific loci influencing pediatric abmd and bmc at the distal radius. Hum. Mol. Genet. 24 (17), 5053–5059. 10.1093/hmg/ddv210 26041818 PMC4527490

[B18] Chu J. Jia J. Yang L. Qu Y. Yin H. Wan J. (2020). Lncrna Mir31hg functions as a cerna to regulate C-met function by sponging mir-34a in esophageal squamous cell carcinoma. Biomed. Pharmacother. = Biomedecine Pharmacother. 128, 110313. Epub 2020/06/06. 10.1016/j.biopha.2020.110313 32502839

[B19] Ciardiello F. Ciardiello D. Martini G. Napolitano S. Tabernero J. Cervantes A. (2022). Clinical management of metastatic colorectal cancer in the era of precision medicine. CA a cancer J. Clin. 72 (4), 372–401. Epub 2022/04/27. 10.3322/caac.21728 35472088

[B20] Clevers H. Nusse R. (2012). Wnt/Β-catenin signaling and disease. Cell 149 (6), 1192–1205. Epub 2012/06/12. 10.1016/j.cell.2012.05.012 22682243

[B21] Corcoran D. L. Pandit K. V. Gordon B. Bhattacharjee A. Kaminski N. Benos P. V. (2009). Features of mammalian microrna promoters emerge from polymerase ii chromatin immunoprecipitation data. PloS one 4 (4), e5279. Epub 2009/04/25. 10.1371/journal.pone.0005279 19390574 PMC2668758

[B22] Deane K. D. Holers V. M. (2021). Rheumatoid arthritis pathogenesis, prediction, and prevention: an emerging paradigm shift. Arthritis Rheumatol. 73 (2), 181–193. 10.1002/art.41417 32602263 PMC7772259

[B23] Daly M. J. Rioux J. D. Schaffner S. F. Hudson T. J. Lander E. S. (2001). High-resolution haplotype structure in the human Genome. Nat. Genet. 29 (2), 229–232. Epub 2001/10/05. 10.1038/ng1001-229 11586305

[B24] Dandan W. Jianliang C. Haiyan H. Hang M. Xuedong L. (2019). Long noncoding rna Mir31hg is activated by Sp1 and promotes cell migration and invasion by sponging mir-214 in nsclc. Gene 692, 223–230. Epub 2019/01/20. 10.1016/j.gene.2018.12.077 30659947

[B25] Das K. Mohanty S. (2017). Hirschsprung disease - current diagnosis and management. Indian J. Pediatr. 84 (8), 618–623. Epub 2017/06/11. 10.1007/s12098-017-2371-8 28600660

[B26] De Simone V. Franzè E. Ronchetti G. Colantoni A. Fantini M. C. Di Fusco D. (2015). Th17-type cytokines, IL-6 and TNF-α synergistically activate STAT3 and NF-kB to promote colorectal cancer cell growth. Oncogene 34 (27), 3493–3503. Epub 2014/09/02. 10.1038/onc.2014.286 25174402 PMC4493653

[B27] Derrien T. Johnson R. Bussotti G. Tanzer A. Djebali S. Tilgner H. (2012). The gencode V7 catalog of human long noncoding rnas: analysis of their gene structure, evolution, and expression. Genome Res. 22 (9), 1775–1789. Epub 2012/09/08. 10.1101/gr.132159.111 22955988 PMC3431493

[B28] Deyo R. A. Mirza S. K. (2016). Clinical practice. Herniated lumbar intervertebral disk. N. Engl. J. Med. 374 (18), 1763–1772. Epub 2016/05/06. 10.1056/NEJMcp1512658 27144851

[B29] Dhamija S. Diederichs S. (2016). From Junk to master regulators of invasion: lncrna functions in migration, emt and metastasis. Int. J. cancer 139 (2), 269–280. Epub 2016/02/16. 10.1002/ijc.30039 26875870

[B30] Ding J. Lu B. Wang J. Wang J. Shi Y. Lian Y. (2015). Long non-coding rna Loc554202 induces apoptosis in colorectal cancer cells via the caspase cleavage cascades. J. Exp. Clin. cancer Res. CR 34 (1), 100. Epub 2015/09/13. 10.1186/s13046-015-0217-7 26362196 PMC4567799

[B31] Eide P. W. Eilertsen I. A. Sveen A. Lothe R. A. (2019). Long noncoding rna Mir31hg is a bona fide prognostic marker with colorectal cancer cell-intrinsic properties. Cancer Manag. Res. 144 (11), 2843–2853. Epub 2019/10/01. 10.1002/ijc.31998 PMC6590447 30447009

[B32] Feng B. Chen K. Zhang W. Zheng Q. He Y. (2022). Silencing of lncrna Mir31hg promotes nasopharyngeal carcinoma cell proliferation and inhibits apoptosis through suppressing the pi3k/akt signaling pathway. J. Clin. Lab. Anal. 36, e24720. 10.1002/jcla.24720 36347827 PMC9757018

[B33] Feng M. B. Li G. H. Dou F. F. (2020). Long-chain non-coding rna Loc554202 promotes proliferation, migration, and invasion of nasopharyngeal carcinoma cells by binding to microrna-31 expression and regulating rhoa expression. Eur. Rev. Med. Pharmacol. Sci. 24 (20), 10550–10556. Epub 2020/11/07. 10.26355/eurrev_202010_23408 33155211

[B34] Gao J. Chen F. Hua M. Guo J. Nong Y. Tang Q. (2018). Knockdown of lncrna Mir31hg inhibits cell proliferation in human hacat keratinocytes. Biol. Res. 51 (1), 30. Epub 2018/09/06. 10.1186/s40659-018-0181-8 30180891 PMC6122774

[B35] Ghafouri-Fard S. Abak A. Tondro Anamag F. Shoorei H. Majidpoor J. Taheri M. (2021). The emerging role of non-coding rnas in the regulation of pi3k/akt pathway in the carcinogenesis process. Biomed. Pharmacother. = Biomedecine Pharmacother. 137, 111279. Epub 2021/01/26. 10.1016/j.biopha.2021.111279 33493969

[B36] Gill J. Gorlick R. (2021). Advancing therapy for osteosarcoma. Nat. Rev. Clin. Oncol. 18 (10), 609–624. 10.1038/s41571-021-00519-8 34131316

[B37] Griffiths C. E. Barker J. N. (2007). Pathogenesis and clinical features of psoriasis. Lancet (London, Engl. 370 (9583), 263–271. Epub 2007/07/31. 10.1016/s0140-6736(07)61128-3 17658397

[B38] Guilloteau K. Paris I. Pedretti N. Boniface K. Juchaux F. Huguier V. (2010). Skin inflammation induced by the synergistic action of il-17a, il-22, oncostatin M, il-1{Alpha}, and Tnf-{Alpha} recapitulates some features of psoriasis. J. Immunol. 184 (9), 5263–5270. Epub 2010/03/26. 10.4049/jimmunol.0902464 20335534

[B39] Guo T. Liu D. Peng S. Wang M. Li Y. (2021). A positive feedback loop of lncrna mir31hg-mir-361-3p -Yy1 accelerates colorectal cancer progression through modulating proliferation, angiogenesis, and glycolysis. Front. Oncol. 11, 684984. Epub 2021/09/07. 10.3389/fonc.2021.684984 34485123 PMC8416113

[B40] Gupta S. C. Awasthee N. Rai V. Chava S. Gunda V. Challagundla K. B. (2020). Long non-coding rnas and nuclear factor-Κb crosstalk in cancer and other human diseases. Biochimica biophysica acta Rev. cancer 1873 (1), 188316. Epub 2019/10/23. 10.1016/j.bbcan.2019.188316 PMC7775411 31639408

[B41] Gyamfi J. Kim J. Choi J. (2022). Cancer as a metabolic disorder. Int. J. Mol. Sci. 23 (3), 1155. Epub 2022/02/16. 10.3390/ijms23031155 35163079 PMC8835572

[B42] Hao L. Wu W. Xu Y. Chen Y. Meng C. Yun J. (2023). Lncrna-Malat1: a key participant in the occurrence and development of cancer. Molecules 28 (5), 2126. 10.3390/molecules28052126 36903369 PMC10004581

[B43] Haunerland N. H. Spener F. (2004). Fatty acid-binding proteins-insights from genetic manipulations. Prog. lipid Res. 43 (4), 328–349. Epub 2004/07/06. 10.1016/j.plipres.2004.05.001 15234551

[B44] He A. Chen Z. Mei H. Liu Y. (2016). Decreased expression of lncrna Mir31hg in human bladder cancer. Cancer Biomark. 17 (2), 231–236. Epub 2016/07/20. 10.3233/cbm-160635 27434291 PMC13020480

[B45] He J. Jin S. Zhang W. Wu D. Li J. Xu J. (2019). Long non-coding rna Loc554202 promotes acquired gefitinib resistance in non-small cell lung cancer through upregulating mir-31 expression. J. Cancer 10 (24), 6003–6013. Epub 2019/11/26. 10.7150/jca.35097 31762810 PMC6856583

[B46] Hu X. Hao D. Yin J. Gong F. Wang X. Wang R. (2022). Association between Mir31hg polymorphisms and the risk of lumbar disc herniation in Chinese han population. Cell cycleGeorget. Tex) 21 (19), 2109–2120. Epub 2022/06/16. 10.1080/15384101.2022.2087281 PMC9467585 35704669

[B47] Huang Y. Jin C. Zheng Y. Li X. Zhang S. Zhang Y. (2017). Knockdown of lncrna Mir31hg inhibits adipocyte differentiation of human adipose-derived stem cells via histone modification of Fabp4. Nat. Commun. 7 (1), 8080. 10.1038/s41598-017-08131-6 PMC5556051 28808264

[B48] Hugo H. Ackland M. L. Blick T. Lawrence M. G. Clements J. A. Williams E. D. (2007). Epithelial-Mesenchymal and mesenchymal-epithelial transitions in carcinoma progression. J. Cell. physiology 213 (2), 374–383. Epub 2007/08/08. 10.1002/jcp.21223 17680632

[B49] Jin C. Jia L. Huang Y. Zheng Y. Du N. Liu Y. (2016). Inhibition of lncrna Mir31hg promotes osteogenic differentiation of human adipose-derived stem cells. Nat. Commun. 34 (11), 2707–2720. 10.1002/stem.2439 27334046

[B50] Kansara M. Teng M. W. Smyth M. J. Thomas D. M. (2014). Translational biology of osteosarcoma. Nat. Rev. Cancer 14 (11), 722–735. Epub 2014/10/17. 10.1038/nrc3838 25319867

[B51] Ko C. C. Hsieh Y. Y. Yang P. M. (2022). Long non-coding rna Mir31hg promotes the transforming growth factor Β-induced epithelial-mesenchymal transition in pancreatic ductal adenocarcinoma cells. Int. J. Mol. Sci. 23 (12), 6559. 10.3390/ijms23126559 35743003 PMC9223781

[B52] Kopp F. Mendell J. T. (2018). Functional classification and experimental dissection of long noncoding rnas. Cell 172 (3), 393–407. Epub 2018/01/27. 10.1016/j.cell.2018.01.011 29373828 PMC5978744

[B53] Kotake Y. Nakagawa T. Kitagawa K. Suzuki S. Liu N. Kitagawa M. (2011). Long non-coding rna anril is required for the Prc2 recruitment to and silencing of P15(ink4b) tumor suppressor gene. Oncogene 30 (16), 1956–1962. Epub 2010/12/15. 10.1038/onc.2010.568 21151178 PMC3230933

[B54] Kuilman T. Michaloglou C. Mooi W. J. Peeper D. S. (2010). The essence of senescence. Genes & Dev. 24 (22), 2463–2479. Epub 2010/11/17. 10.1101/gad.1971610 21078816 PMC2975923

[B55] LaGory E. L. Giaccia A. J. (2016). The ever-expanding role of hif in tumour and stromal biology. Nat. Cell Biol. 18 (4), 356–365. Epub 2016/03/31. 10.1038/ncb3330 27027486 PMC4898054

[B56] Langer J. C. (2013). Hirschsprung disease. Curr. Opin. Pediatr. 25 (3), 368–374. Epub 2013/04/26. 10.1097/MOP.0b013e328360c2a0 23615177

[B57] Lenis A. T. Lec P. M. Chamie K. Mshs M. D. (2020). Bladder cancer: a review. Jama 324 (19), 1980–1991. Epub 2020/11/18. 10.1001/jama.2020.17598 33201207

[B58] Li N. Zhan X. (2019). Identification of clinical trait-related lncrna and mrna biomarkers with weighted gene Co-expression network analysis as useful tool for personalized medicine in ovarian cancer. J. Cell. Biochem. 10 (3), 273–290. 10.1007/s13167-019-00175-0 PMC6695468 31462944

[B59] Li Y. (2020). Mir31hg exhibits oncogenic property and acts as a sponge for mir-361-3p in cervical carcinoma. Biochem. biophysical Res. Commun. 529 (4), 890–897. Epub 2020/08/21. 10.1016/j.bbrc.2020.06.028 32819595

[B60] Li Y. Xin S. Wu H. Xing C. Duan L. Sun W. (2018). High expression of microrna-31 and its host gene Loc554202 predict favorable outcomes in patients with colorectal cancer treated with oxaliplatin. Oncol. Rep. 40 (3), 1706–1724. Epub 2018/07/18. 10.3892/or.2018.6571 30015936

[B61] Lin Y. Zhang C. S. Li S. J. Li Z. Sun F. B. (2018). Lncrna Loc554202 promotes proliferation and migration of gastric cancer cells through regulating P21 and E-cadherin. Eur. Rev. Med. Pharmacol. Sci. 22 (24), 8690–8697. Epub 2018/12/24. 10.26355/eurrev_201812_16634 30575909

[B62] Liu H. Li D. Sun L. Qin H. Fan A. Meng L. (2022b). Interaction of lncrna Mir100hg with Hnrnpa2b1 facilitates M(6)a-dependent stabilization of Tcf7l2 mrna and colorectal cancer progression. Mol. cancer 21 (1), 74. Epub 2022/03/14. 10.1186/s12943-022-01555-3 35279145 PMC8917698

[B63] Liu W. Wang X. Chen J. Zeng F. Xiong J. (2022a). The polymorphisms of Mir31hg gene is correlated with alcohol-induced osteonecrosis of the femoral head in Chinese han male population. Front. Endocrinol. 13, 976165. Epub 2022/12/13. 10.3389/fendo.2022.976165 PMC9731210 36506078

[B64] Lu W. Cao F. Feng L. Song G. Chang Y. Chu Y. (2021). Lncrna Snhg6 regulates the differentiation of mdscs by regulating the ubiquitination of Ezh2. J. Hematol. Oncol. 14 (1), 196. 10.1186/s13045-021-01212-0 34794493 PMC8600792

[B65] Ma X. Qi S. Duan Z. Liao H. Yang B. Wang W. (2017). Long non-coding rna Loc554202 modulates chordoma cell proliferation and invasion by recruiting Ezh2 and regulating mir-31 expression. Cell Prolif. 50 (6), e12388. 10.1111/cpr.12388 28963737 PMC6529120

[B66] Meltzer P. S. Helman L. J. (2021). New horizons in the treatment of osteosarcoma. N. Engl. J. Med. 385 (22), 2066–2076. Epub 2021/11/25. 10.1056/NEJMra2103423 34818481

[B67] Mo X. Hu D. Yang P. Li Y. Bashir S. Nai A. (2022). A novel cuproptosis-related prognostic lncrna signature and lncrna mir31hg/mir-193a-3p/tnfrsf21 regulatory Axis in lung adenocarcinoma. Front. Oncol. 12, 927706. Epub 2022/08/09. 10.3389/fonc.2022.927706 35936736 PMC9353736

[B68] Mody M. D. Rocco J. W. Yom S. S. Haddad R. I. Saba N. F. (2021). Head and neck cancer. Lancet (London, Engl. 398 (10318), 2289–2299. Epub 2021/09/26. 10.1016/s0140-6736(21)01550-6 34562395

[B69] Montes M. Lubas M. Arendrup F. S. Mentz B. Rohatgi N. Tumas S. (2021). The long non-coding rna Mir31hg regulates the senescence associated secretory phenotype. Nat. Commun. 12 (1), 2459. 10.1038/s41467-021-22746-4 33911076 PMC8080841

[B70] Montes M. Nielsen M. M. Maglieri G. Jacobsen A. Højfeldt J. Agrawal-Singh S. (2015). The lncrna Mir31hg regulates P16(ink4a) expression to modulate senescence. Nat. Commun. 6, 6967. 10.1038/ncomms7967 25908244

[B71] Mundi P. S. Sachdev J. McCourt C. Kalinsky K. (2016). Akt in cancer: new molecular insights and advances in drug development. Br. J. Clin. Pharmacol. 82 (4), 943–956. Epub 2016/05/28. 10.1111/bcp.13021 27232857 PMC5137819

[B72] Nassar K. W. Tan A. C. (2020). The mutational landscape of mucosal melanoma. Seminars cancer Biol. 61, 139–148. Epub 2019/10/28. 10.1016/j.semcancer.2019.09.013 PMC7078020 31655118

[B73] Nie F. Q. Ma S. Xie M. Liu Y. W. De W. Liu X. H. (2016). Decreased long noncoding rna Mir31hg is correlated with poor prognosis and contributes to cell proliferation in gastric cancer. Tumour Biol. J. Int. Soc. Oncodevelopmental Biol. Med. 37 (6), 7693–7701. Epub 2015/12/23. 10.1007/s13277-015-4644-z 26692098

[B74] Pattrapornpisut P. Avila-Casado C. Reich H. N. (2021). Iga nephropathy: core curriculum 2021. Am. J. kidney Dis. official J. Natl. Kidney Found. 78 (3), 429–441. Epub 2021/07/13. 10.1053/j.ajkd.2021.01.024 34247883

[B75] Peng S. Chen L. Yuan Z. Duan S. (2022). Suppression of Mir31hg affects the functional properties of thyroid cancer cells depending on the mir-761/mapk1 Axis. BMC Endocr. Disord. 22 (1), 107. Epub 2022/04/22. 10.1186/s12902-022-00962-3 35443670 PMC9022350

[B76] Petek D. Hannouche D. Suva D. (2019). Osteonecrosis of the femoral head: pathophysiology and current concepts of treatment. EFORT open Rev. 4 (3), 85–97. Epub 2019/04/18. 10.1302/2058-5241.4.180036 30993010 PMC6440301

[B77] Puvvula P. K. Desetty R. D. Pineau P. Marchio A. Moon A. Dejean A. (2014). Long noncoding rna panda and scaffold-attachment-factor safa control senescence entry and exit. Nat. Commun. 5, 5323. Epub 2014/11/20. 10.1038/ncomms6323 25406515 PMC4263151

[B78] Qin J. Ning H. Zhou Y. Hu Y. Yang L. Huang R. (2018). Lncrna Mir31hg overexpression serves as poor prognostic biomarker and promotes cells proliferation in lung adenocarcinoma. Biomed. Pharmacother. = Biomedecine Pharmacother. 99, 363–368. Epub 2018/01/26. 10.1016/j.biopha.2018.01.037 29367106

[B79] Ren Z. P. Chu X. Y. Xue Z. Q. Zhang L. B. Wen J. X. Deng J. Q. (2017). Down-regulation of lncrna Mir31hg correlated with aggressive clinicopathological features and unfavorable prognosis in esophageal squamous cell carcinoma. Eur. Rev. Med. Pharmacol. Sci. 21 (17), 3866–3870. 28975978

[B80] Richart L. Picod-Chedotel M. L. Wassef M. Macario M. Aflaki S. Salvador M. A. (2022). Xist loss impairs mammary stem cell differentiation and increases tumorigenicity through mediator hyperactivation. Cell 185 (12), 2164–2183.e25. e25. Epub 2022/05/22. 10.1016/j.cell.2022.04.034 35597241

[B81] Roberts T. C. Morris K. V. Weinberg M. S. (2014). Perspectives on the mechanism of transcriptional regulation by long non-coding rnas. Epigenetics 9 (1), 13–20. Epub 2013/10/24. 10.4161/epi.26700 24149621 PMC3928176

[B82] Saha M. K. Julian B. A. Novak J. Rizk D. V. (2018). Secondary iga nephropathy. Kidney Int. 94 (4), 674–681. Epub 2018/05/29. 10.1016/j.kint.2018.02.030 29804660 PMC6981247

[B83] Shi Y. Lu J. Zhou J. Tan X. He Y. Ding J. (2014). Long non-coding rna Loc554202 regulates proliferation and migration in breast cancer cells. Biochem. biophysical Res. Commun. 446 (2), 448–453. Epub 2014/03/19. 10.1016/j.bbrc.2014.02.144 24631686

[B84] Shih J. W. Chiang W. F. Wu A. T. H. Wu M. H. Wang L. Y. Yu Y. L. (2017). Long noncoding rna lnchifcar/mir31hg is a hif-1α Co-activator driving oral cancer progression. EPMA J. 8, 15874. 10.1038/ncomms15874 PMC5489688 28639619

[B85] Sinicrope F. A. (2022). Increasing incidence of early-onset colorectal cancer. N. Engl. J. Med. 386 (16), 1547–1558. Epub 2022/04/21. 10.1056/NEJMra2200869 35443109

[B86] Smolen J. S. Aletaha D. McInnes I. B. (2016). Rheumatoid arthritis. Lancet (London, Engl. 388 (10055), 2023–2038. Epub 2016/10/30. 10.1016/s0140-6736(16)30173-8 27156434

[B87] Storer M. Mas A. Robert-Moreno A. Pecoraro M. Ortells M. C. Di Giacomo V. (2013). Senescence is a developmental mechanism that contributes to embryonic growth and patterning. Cell 155 (5), 1119–1130. Epub 2013/11/19. 10.1016/j.cell.2013.10.041 24238961

[B88] Stratikopoulos E. E. Parsons R. E. (2016). Molecular pathways: targeting the Pi3k pathway in cancer-bet inhibitors to the rescue. Clin. cancer Res. official J. Am. Assoc. Cancer Res. 22 (11), 2605–2610. Epub 2016/06/03. 10.1158/1078-0432.ccr-15-2389 PMC4896088 27250929

[B89] Sun K. Zhao X. Wan J. Yang L. Chu J. Dong S. (2018). The diagnostic value of long non-coding rna Mir31hg and its role in esophageal squamous cell carcinoma. Life Sci. 202, 124–130. Epub 2018/04/02. 10.1016/j.lfs.2018.03.050 29605445

[B90] Sun Y. Jia X. Wang M. Deng Y. (2019). Long noncoding rna Mir31hg abrogates the availability of tumor suppressor microrna-361 for the growth of osteosarcoma. Cancer Manag. Res. 11, 8055–8064. 10.2147/cmar.s214569 31564967 PMC6722458

[B91] Sung H. Ferlay J. Siegel R. L. Laversanne M. Soerjomataram I. Jemal A. (2021). Global cancer statistics 2020: globocan estimates of incidence and mortality worldwide for 36 cancers in 185 countries. CA Cancer J. Clin. 71 (3), 209–249. 10.3322/caac.21660 33538338

[B92] Sveen A. Lothe R. A. Wu S. Nitschke K. Worst T. S. Fierek A. (2020). Long noncoding rna Mir31hg and its splice variants regulate proliferation and migration: prognostic implications for muscle invasive bladder cancer. Int. J. cancer 39 (1), 288. 10.1186/s13046-020-01795-5 PMC7745499 33334367

[B93] Tapp H. Hanley E. N. Jr. Patt J. C. Gruber H. E. (2009). Adipose-derived stem cells: characterization and current application in orthopaedic tissue repair. Exp. Biol. Med. (Maywood, NJ) 234 (1), 1–9. Epub 2008/12/26. 10.3181/0805/mr-170 19109553

[B94] Thai A. A. Solomon B. J. Sequist L. V. Gainor J. F. Heist R. S. (2021). Lung cancer. Lancet (London, Engl. 398, 535–554. Epub 2021/07/18. 10.1016/s0140-6736(21)00312-3 34273294

[B95] Thiery J. P. (2002). Epithelial-mesenchymal transitions in tumour progression. Nat. Rev. Cancer 2 (6), 442–454. Epub 2002/08/22. 10.1038/nrc822 12189386

[B96] Thiery J. P. Sleeman J. P. (2006). Complex networks orchestrate epithelial-mesenchymal transitions. Nat. Rev. Mol. Cell Biol. 7 (2), 131–142. Epub 2006/02/24. 10.1038/nrm1835 16493418

[B97] Thrift A. P. El-Serag H. B. (2020). Burden of gastric cancer. Clin. gastroenterology hepatology official Clin. Pract. J. Am. Gastroenterological Assoc. 18 (3), 534–542. Epub 2019/07/31. 10.1016/j.cgh.2019.07.045 PMC8859863 31362118

[B98] Tu C. Ren X. He J. Li S. Qi L. Duan Z. (2020). The predictive value of lncrna Mir31hg expression on clinical outcomes in patients with solid malignant tumors. Oral Dis. 20, 115. 10.1186/s12935-020-01194-y PMC7137300 32280307

[B99] Tu H. F. Liu C. J. Hung W. W. Shieh T. M. (2022). Co-upregulation of mir-31 and its host gene lncrna Mir31hg in oral squamous cell carcinoma. J. Dent. Sci. 17 (2), 696–706. Epub 2022/06/28. 10.1016/j.jds.2021.11.006 35756773 PMC9201660

[B100] Tu J. Hong W. Zhang P. Wang X. Körner H. Wei W. (2018). Ontology and function of fibroblast-like and macrophage-like synoviocytes: how do they talk to each other and can they Be targeted for rheumatoid arthritis therapy? Front. Immunol. 9, 1467. Epub 2018/07/13. 10.3389/fimmu.2018.01467 29997624 PMC6028561

[B101] Vasan N. Baselga J. Hyman D. M. (2019). A view on drug resistance in cancer. Nature 575 (7782), 299–309. Epub 2019/11/15. 10.1038/s41586-019-1730-1 31723286 PMC8008476

[B102] Vervoort S. J. Devlin J. R. Kwiatkowski N. Teng M. Gray N. S. Johnstone R. W. (2022). Targeting transcription cycles in cancer. Nat. Rev. Cancer 22 (1), 5–24. 10.1038/s41568-021-00411-8 34675395

[B103] Walter K. (2022). Psoriasis. Jama 327 (19), 1936. Epub 2022/05/18. 10.1001/jama.2022.5270 35579640

[B104] Wang A. Ren M. Wang J. (2018b). The pathogenesis of steroid-induced osteonecrosis of the femoral head: a systematic review of the literature. Gene 671, 103–109. Epub 2018/06/03. 10.1016/j.gene.2018.05.091 29859289

[B105] Wang B. Jiang H. Wang L. Chen X. Wu K. Zhang S. (2017). Increased Mir31hg lncrna expression increases gefitinib resistance in non-small cell lung cancer cell lines through the egfr/pi3k/akt signaling pathway. Oncol. Lett. 13 (5), 3494–3500. Epub 2017/05/23. 10.3892/ol.2017.5878 28529576 PMC5431660

[B106] Wang J. Liu B. Cao J. Zhao L. Wang G. (2022b). Mir31hg expression predicts poor prognosis and promotes colorectal cancer progression. Cancer Manag. Res. 14, 1973–1986. Epub 2022/06/24. 10.2147/cmar.s351928 35733512 PMC9208482

[B107] Wang K. C. Chang H. Y. (2011). Molecular mechanisms of long noncoding rnas. Mol. Cell 43 (6), 904–914. Epub 2011/09/20. 10.1016/j.molcel.2011.08.018 21925379 PMC3199020

[B108] Wang R. Ma Z. Feng L. Yang Y. Tan C. Shi Q. (2018a). Lncrna Mir31hg targets Hif1a and P21 to facilitate head and neck cancer cell proliferation and tumorigenesis by promoting cell-cycle progression. Mol. cancer 17 (1), 162. Epub 2018/11/22. 10.1186/s12943-018-0916-8 30458787 PMC6247607

[B109] Wang X. Dong K. Jin Q. Ma Y. Yin S. Wang S. (2019). Upregulation of lncrna Fer1l4 suppresses the proliferation and migration of the hepatocellular carcinoma via regulating pi3k/akt signal pathway. J. Cell Biochem. 120 (4), 6781–6788. 10.1002/jcb.27980 30382631

[B110] Wang X. Wang C. Guan J. Chen B. Xu L. Chen C. (2021a). Progress of breast cancer basic research in China. Int. J. Biol. Sci. 17 (8), 2069–2079. Epub 2021/06/17. 10.7150/ijbs.60631 34131406 PMC8193257

[B111] Wang X. Zhao D. Xie H. Hu Y. (2021b). Interplay of long non-coding rnas and hif-1α: a new dimension to understanding hypoxia-regulated tumor growth and metastasis. Cancer Lett. 499, 49–59. Epub 2020/11/21. 10.1016/j.canlet.2020.11.007 33217445

[B112] Wang Y. Wang Y. Liang D. Hu H. Li G. Meng X. (2022a). Mir31hg polymorphisms are related to steroid-induced osteonecrosis of femoral head among Chinese han population. BMC Musculoskelet. Disord. 23 (1), 836. Epub 2022/09/04. 10.1186/s12891-022-05785-w 36057712 PMC9440494

[B113] Wei Y. Wang X. Zhang Z. Zhao C. Chang Y. Bian Z. (2023). Impact of Mir31hg polymorphisms on risk of breast cancer in Chinese women. Int. J. Clin. Oncol. 28 (5), 664–679. Epub 2023/03/09. 10.1007/s10147-023-02323-z 36884100

[B114] Wu M. Sun J. Wang L. Wang P. Xiao T. Wang S. (2022). The lncrna hotair via mir-17-5p is involved in arsenite-induced hepatic fibrosis through regulation of Th17 cell differentiation. J. Hazard. Mater. 443, 130276. Epub 2022/11/05. 10.1016/j.jhazmat.2022.130276 36332283

[B115] Wu W. Hu Q. Nie E. Yu T. Wu Y. Zhi T. (2017). Hypoxia induces H19 expression through direct and indirect hif-1α activity, promoting oncogenic effects in glioblastoma. Sci. Rep. 7, 45029. Epub 2017/03/23. 10.1038/srep45029 28327666 PMC5361208

[B116] Xin C. Bi X. Xiao C. Dong L. (2021). Mir31hg regulates the proliferation, migration and invasion of breast cancer by regulating the expression of Poldip2. J. buon 26 (2), 459–465. Epub 2021/06/03. 34076993

[B117] Xu H. L. Tian F. Z. (2020). Clinical significance of lncrna Mir31hg in melanoma. Eur. Rev. Med. Pharmacol. Sci. 24 (8), 4389–4395. Epub 2020/05/07. 10.26355/eurrev_202004_21020 32373976

[B118] Yan S. Tang Z. Chen K. Liu Y. Yu G. Chen Q. (2018). Long noncoding rna Mir31hg inhibits hepatocellular carcinoma proliferation and metastasis by sponging microrna-575 to modulate St7l expression. J. Exp. Clin. cancer Res. CR 37 (1), 214. Epub 2018/09/05. 10.1186/s13046-018-0853-9 30176933 PMC6122648

[B119] Yang H. Liu P. Zhang J. Peng X. Lu Z. Yu S. (2016b). Long noncoding rna Mir31hg exhibits oncogenic property in pancreatic ductal adenocarcinoma and is negatively regulated by mir-193b. Oncogene 35 (28), 3647–3657. Epub 2015/11/10. 10.1038/onc.2015.430 26549028 PMC4947634

[B120] Yang L. Deng W. L. Zhao B. G. Xu Y. Wang X. W. Fang Y. (2022b). Foxo3-Induced lncrna Loc554202 contributes to hepatocellular carcinoma progression via the mir-485-5p/bsg Axis. Cancer Gene Ther. 29 (3-4), 326–340. 10.1038/s41417-021-00312-w 33654226 PMC8940625

[B121] Yang L. Wei H. Xiao H. J. (2016a). Long non-coding rna Loc554202 expression as a prognostic factor in patients with colorectal cancer. Eur. Rev. Med. Pharmacol. Sci. 20 (20), 4243–4247. Epub 2016/11/11. 27831651

[B122] Yang M. Lu H. Liu J. Wu S. Kim P. Zhou X. (2022a). Lncrnafunc: a knowledgebase of lncrna function in human cancer. Nucleic Acids Res. 50 (D1), D1295–D1306. 10.1093/nar/gkab1035 34791419 PMC8728133

[B123] Yang S. Wang J. Ge W. Jiang Y. (2018). Long non-coding rna Loc554202 promotes laryngeal squamous cell carcinoma progression through regulating mir-31. J. Cell Biochem. 119 (8), 6953–6960. 10.1002/jcb.26902 29737563

[B124] Yang Y. M. Hong P. Xu W. W. He Q. Y. Li B. (2020). Advances in targeted therapy for esophageal cancer. Signal Transduct. Target Ther. 5 (1), 229. 10.1038/s41392-020-00323-3 33028804 PMC7542465

[B125] Yeoh K. G. Tan P. (2022). Mapping the genomic diaspora of gastric cancer. Nat. Rev. Cancer 22 (2), 71–84. 10.1038/s41568-021-00412-7 34702982

[B126] Yuan H. Li S. Wang L. Zhao X. Xue L. Lei X. (2020). Genetic variants of the Mir31hg gene are related to a risk of iga nephropathy. Int. Immunopharmacol. 84, 106533. Epub 2020/04/29. 10.1016/j.intimp.2020.106533 32344354

[B127] Zhang B. Xu H. Wang J. Liu B. Sun G. (2017b). A narrative review of non-operative treatment, especially traditional Chinese medicine therapy, for lumbar intervertebral disc herniation. Biosci. trends 11 (4), 406–417. Epub 2017/09/15. 10.5582/bst.2017.01199 28904328

[B128] Zhang R. Wu D. Wang Y. Wu L. Gao G. Shan D. (2021). Withdrawn: lncRNA MIR31HG is activated by STAT1 and facilitates glioblastoma cell growth via wnt/β-catenin signaling pathway. Neurosci. Res. Epub 2021/05/04. 10.1016/j.neures.2021.04.008 33940081

[B129] Zhang Y. Alexander P. B. Wang X. F. (2017a). Tgf-Β family signaling in the control of cell proliferation and survival. Cold Spring Harb. Perspect. Biol. 9 (4), a022145. Epub 2016/12/07. 10.1101/cshperspect.a022145 27920038 PMC5378054

[B130] Zheng S. Zhang X. Wang X. Li J. (2019). Mir31hg promotes cell proliferation and invasion by activating the wnt/Β-catenin signaling pathway in non-small cell lung cancer. Oncol. Lett. 17 (1), 221–229. Epub 2019/01/19. 10.3892/ol.2018.9607 30655759 PMC6313218

[B131] Zhou C. Ye L. Jiang C. Bai J. Chi Y. Zhang H. (2015). Long noncoding rna hotair, a hypoxia-inducible factor-1α activated driver of malignancy, enhances hypoxic cancer cell proliferation, migration, and invasion in non-small cell lung cancer. Tumour Biol. J. Int. Soc. Oncodevelopmental Biol. Med. 36 (12), 9179–9188. Epub 2015/06/20. 10.1007/s13277-015-3453-8 26088446

[B132] Zhou T. Lin K. Nie J. Pan B. He B. Pan Y. (2021). Lncrna spint1-as1 promotes breast cancer proliferation and metastasis by sponging let-7 a/B/I-5p. Pathology, Res. Pract. 217, 153268. Epub 2020/11/28. 10.1016/j.prp.2020.153268 33246290

